# Non-destructive Phenotyping to Identify *Brachiaria* Hybrids Tolerant to Waterlogging Stress under Field Conditions

**DOI:** 10.3389/fpls.2017.00167

**Published:** 2017-02-13

**Authors:** Juan de la Cruz Jiménez, Juan A. Cardoso, Luisa F. Leiva, Juanita Gil, Manuel G. Forero, Margaret L. Worthington, John W. Miles, Idupulapati M. Rao

**Affiliations:** ^1^School of Plant Biology, The University of Western Australia, CrawleyWA, Australia; ^2^International Center for Tropical AgricultureCali, Colombia; ^3^Semillero de Investigación LÚN, Grupo D+TEC, Universidad de IbaguéIbagué, Colombia; ^4^Department of Horticulture, University of Arkansas, FayettevilleAR, USA

**Keywords:** image analysis, image segmentation, NDVI, tropical grasses, soil flooding

## Abstract

*Brachiaria* grasses are sown in tropical regions around the world, especially in the Neotropics, to improve livestock production. Waterlogging is a major constraint to the productivity and persistence of *Brachiaria* grasses during the rainy season. While some *Brachiaria* cultivars are moderately tolerant to seasonal waterlogging, none of the commercial cultivars combines superior yield potential and nutritional quality with a high level of waterlogging tolerance. The *Brachiaria* breeding program at the International Center for Tropical Agriculture, has been using recurrent selection for the past two decades to combine forage yield with resistance to biotic and abiotic stress factors. The main objective of this study was to test the suitability of normalized difference vegetation index (NDVI) and image-based phenotyping as non-destructive approaches to identify *Brachiaria* hybrids tolerant to waterlogging stress under field conditions. Nineteen promising hybrid selections from the breeding program and three commercial checks were evaluated for their tolerance to waterlogging under field conditions. The waterlogging treatment was imposed by applying and maintaining water to 3 cm above soil surface. Plant performance was determined non-destructively using proximal sensing and image-based phenotyping and also destructively via harvesting for comparison. Image analysis of projected green and dead areas, NDVI and shoot biomass were positively correlated (*r* ≥ 0.8). Our results indicate that image analysis and NDVI can serve as non-destructive screening approaches for the identification of *Brachiaria* hybrids tolerant to waterlogging stress.

## Introduction

Due to climate change, it is expected that extreme rainfall events will increase in some parts of the tropics in the future (Hirabayashi et al., 2013). This projected increase in precipitation over shorter periods of time will likely result in temporarily waterlogged soil conditions. Waterlogging causes a hypoxic or anoxic soil environment due to the slow diffusion of oxygen between the atmosphere and waterlogged soil, and by the rapid consumption of O_2_ by microorganisms and roots (Ponnamperuma, 1972; Colmer, 2003; Koppitz, 2004; Colmer and Voesenek, 2009; Unger et al., 2009). Oxygen deficiency in waterlogged soil limits root aerobic respiration (Jackson and Drew, 1984). As a result, symptoms such as leaf senescence and reduced or stunted growth are common in plants not adapted to waterlogged conditions (Malik et al., 2015).

Livestock production is one of the main economic activities across Latin America (Rivas and Holmann, 2004). Livestock productivity depends largely on forage productivity (Boval and Dixon, 2012). *Brachiaria* grasses are the most widely sown forages in tropical Latin America, and their productivity and persistence are affected by waterlogging (Baruch, 1994; Rao, 2014). Plant breeding has the potential to improve waterlogging tolerance in *Brachiaria* grasses since inter-and intraspecific variation has been found (Cardoso et al., 2014a,b). The International Center for Tropical Agriculture (CIAT) conducts a *Brachiaria* breeding program which aims to develop interspecific *Brachiaria* hybrids with greater forage and seed yield, improved forage quality, and adaptation to biotic and abiotic stress factors (including waterlogging tolerance) (Miles, 2007).

Shoot biomass (i.e., forage yield) is one of the most important target traits for improvement in any forage breeding program. However, measurement of shoot biomass is a labor and time consuming process that involves a significant amount of sample processing (harvesting, drying, and weighing) (Schaefer and Lamb, 2016). Plant height is commonly measured to estimate shoot biomass, particularly in forage grasses with erect growth habit (Harmoney et al., 1997; Ganguli et al., 2000). However, plant height does not provide information on the green vs. senescent or chlorotic (from now on referred to as ‘dead’) fraction of shoot biomass. Such distinction between fractions of the shoot biomass in *Brachiaria* hybrids is relevant because the green fraction contributes to the potential productivity of the hybrid, whereas the dead fraction indicates the sensitivity of the hybrid to a number of environmental stresses, including waterlogging (Cardoso et al., 2013; Rao, 2014), and influences forage quality. Yet, distinction between green and dead fractions of the shoot biomass is not often applied in field studies due to labor and time constraints.

Visual evaluation of symptoms (green vs. dead fraction of shoot) under waterlogging stress has traditionally been used to evaluate the tolerance of selected *Brachiaria* hybrids in the CIAT breeding program. However, such evaluations are laborious, often biased by the examiner and may not be sufficiently accurate (Walter et al., 2012). In the face of such limitations, there is increasing interest in implementing non-destructive approaches to evaluate *Brachiaria* hybrids under waterlogging conditions, particularly applying techniques that were developed for use under field conditions and are currently tested on other major crops (e.g., Rundquist et al., 2004; Scotford and Miller, 2004; Montes et al., 2007; Lan et al., 2009; McCarthy et al., 2010; Comar et al., 2012; White et al., 2012; White and Conley, 2013; Andrade-Sanchez et al., 2014; Araus and Cairns, 2014; Deery et al., 2014; Barker et al., 2016; Lootens et al., 2016). The use of non-destructive automated phenotyping may enable more accurate quantification of symptoms, representing a strong possibility to achieve progress in genetic gain (Walter et al., 2012).

One such technique is the use of the normalized difference vegetation index (NDVI). The NDVI measures differences of reflectance in the red and near-infrared regions of the spectrum and provides an estimation of plant biomass and senescence (Di Bella et al., 2004; Verhulst and Govaerts, 2010). The leaves of a healthy plant absorb more red light and reflect more of the near infrared light, resulting in high NDVI values. As a result, NDVI handheld sensors were developed to monitor plant health (Verhulst and Govaerts, 2010) and currently are widely used for screening purposes for resistance to abiotic stress (Lopes and Reynolds, 2012; Adebayo et al., 2014; Arguello et al., 2016). More recently, digital images have been used to assess plant responses to different abiotic constraints (for examples see, Granier et al., 2006; Tackenberg, 2007; Walter et al., 2007; Golzarian et al., 2011; Hartmann et al., 2011; Humplík et al., 2015a,b).

Each of these techniques (NDVI and digital photography) have disadvantages but might be complimentary to each other. A common set-up for the non-destructive and proximal sensing of plants under field conditions (using NDVI and digital images) relies on data acquisition from the top of the plant. An overhead digital image can discriminate between green and dead fractions (Camargo et al., 2014), but cannot predict plant height. Whereas, NDVI might provide an estimate of plant height and shoot biomass (Payero et al., 2004; Pittman et al., 2015) but cannot separate green from dead fractions of the shoot biomass. Therefore, a comprehensive understanding of plant performance under waterlogging conditions could be reached by using both NDVI and digital photography. For that reason, the main objective of the present study was to test NDVI and digital images as complementary non-destructive approaches to identify *Brachiaria* hybrids tolerant to waterlogging stress under field conditions.

## Materials and Methods

### Plant Material and Growing Conditions

The field trial was conducted in July, 2015 at the CIAT headquarters in Palmira, Colombia (lat. 3° 29′ N; long. 76° 21′ W; altitude 965 m). Plants grown in a Mollisol (Fluventic Haplustept), clay-loam, pH 7.8, organic matter 18.4 g/kg and bulk density 1.6 g/cm^3^. The genotypes used in this study were 19 hybrids generated from interspecific crosses (*B. brizantha* × *B. decumbens* × *B. ruziziensis*) in the CIAT *Brachiaria* breeding program as well as 3 commercial cultivars (*B. decumbens* cv. Basilisk [CIAT 606]; *B. brizantha* cv. Marandu [CIAT 6294]; and the interspecific *Brachiaria* hybrid cv. Caymán [BR02/1752]) as checks. The 19 test hybrids were selected from a larger group of advanced hybrid breeding materials based on their high biomass production after 2 weeks of continuous waterlogging in a previous pot trial (data not shown). The trial was performed as a split plot experiment with control and waterlogged treatments assigned to main plots (8 m width × 36 m long) organized in a randomized complete block design with two replicates and the 22 genotypes assigned to subplots in a randomized complete block design with six replicates per main plot.

Vegetative propagules of each genotype were planted in Jiffy mix (Carefree^®^, Jiffy International, Norway) for an establishment period of 2 weeks before being transplanted into the field. Plants were then allowed to grow for an additional period of 2 months under field conditions spaced by 2 m before the experimental treatments were imposed. After the establishment period, waterlogged and control treatments were imposed for 2 weeks. Main plots were leveled to 0° and surrounded by a compacted berms (50 cm height and 60 cm length). The waterlogged treatment was imposed by applying water to a lamina of three centimeters above soil surface by flood irrigation. Waterlogged plots were irrigated daily in order to maintain the water level during the entire 2-week treatment period. Control plots were supplied with adequate water at the beginning of the treatment and the water table was monitored using homemade piezometers. Average temperature was 31/20°C (day/night) and total precipitation was 1.5 mm during the 2 week treatment period.

### Conventional Destructive Phenotyping

Initial vigor (IV) before waterlogging treatment was assessed using a five-point, visual scale: level ‘5’ indicated high shoot growth with many leaves and tillers while level ‘1’ represented low shoot growth with fewer tillers and less leaf growth. A second visual evaluation (VE) at harvest was performed only in waterlogged plants. This VE consisted of a waterlogging injury score that was used to characterize the performance of the plants after waterlogging conditions: level ‘5’ represented plants without any visible damage and ‘1’ denoted dead plants. Plant height (PH) was measured manually using a ruler before starting waterlogging treatment and at harvest.

After 2 weeks of treatment, aboveground biomass was harvested at 1 cm above soil surface and oven dried at 60°C for 3 days. Finally, aboveground tissue including leaves and stems were weighed and expressed as shoot biomass (SHB). Then SHB components were separated into dead leaf biomass (DLB) and green leaf biomass (GLB). The waterlogging tolerance coefficient (WTC) was determined according to Liu et al. (2010) and expressed in percentage:

$$\begin{array}{c} \mathrm{WTC}=\frac{\mathrm{Shoot}\;\mathrm{biomas}\;\mathrm{under}\;\mathrm{waterlogged}\;\mathrm{conditions}\;(g.{\mathrm{plant}}^{-1})}{\mathrm{Shoot}\;\mathrm{biomas}\;\mathrm{under}\;\mathrm{control}\;\mathrm{conditions}\;(g.{\mathrm{plant}}^{-1})}\times 100 \end{array}$$

### NDVI and Digital Image Processing for Non-destructive Phenotyping

**FIGURE 1:**
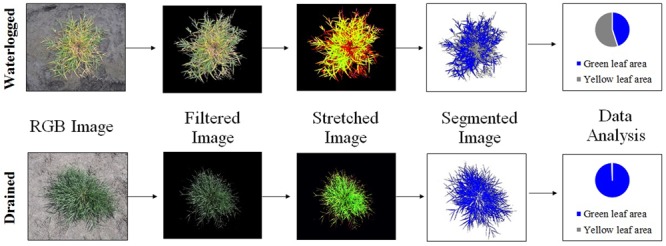
**Work flow of image analysis steps used in the calculation of projected green and dead plant biomass from overhead digital photography**.

Digital color images of each plant were taken individually before imposing treatments and at harvest from 1.5 m above the soil surface using a Coolpix P6000 camera (Nikon, Japan) with a resolution of 13 megapixels. Digital images were saved in 4224 × 3168 pixel JPG format and analyzed by a set of instructions written in Java and run in ImageJ software (National Institutes of Health, USA). A workflow of the image analysis program is summarized in **Figure 1**. The main steps of image analysis were performed to classify pixels of the image into its components, namely soil, green leaves, and dead leaves as follows. (1) Image background information was eliminated by using a previous color threshold learning adjusted to remove soil pixels from the image. This process outputs plant silhouettes without background noise. (2) A stretching process was applied to increase color contrast and to reduce correlation among similar pixels. This process enhances subtle differences in hue by creating a false image which will be easily segmented afterward. (3) Images were segmented by a pattern recognition approach using a modified K-means classifier. This non-supervised classifier used Euclidian distance to classify pixels clusters according to classes of interest, e.g., dead leaves vs. green leaves. (4) Segmented pictures were analyzed by counting pixels according to different regions of interest. (5) Data were recorded in.csv format and expressed in percentage of pixels according to different areas of interest in the original picture. For instance, green pixels refers to green areas in the picture and yellow pixels refers to both chlorotic and senescent tissues in the picture. Finally, the number of pixels in each region of interest was converted to mm^2^. Immediately before the conventional destructive phenotyping, we measured the NDVI from 1.5 m above the soil surface using a hand-held device (GreenSeeker^®^, Trimble, USA). Projected WTC was also determined following the methodology previously used in the section before and comparing projected green areas under waterlogging and control conditions.

### Statistical Analysis

Statistical analyses were conducted using SAS 9.2 (SAS Institute, Inc., USA). Analyses of variance (ANOVA) were performed to test for the effect of treatments and traits measured. Least-square means were calculated for all traits using PROC MIXED, genotypes and treatments were considered as fixed effects and replications as random effects. Least-square means were then used to calculate Pearson’s correlations. Means of measured traits, variation coefficient and Fisher’s protected LSD were also calculated to identify differences among treatments and genotypes.

## Results

Big differences in green leaf biomass and dead leaf biomass produced under waterlogging conditions were prominent across evaluated hybrids, indicating that sufficient genetic variation for waterlogging tolerance exists within the CIAT *Brachiaria* breeding program and that current screening methodology can be used to effectively select for increased tolerance to waterlogging. We found that both conventional and non-destructive phenotyping can be used to accurately select *Brachiaria* hybrids under waterlogged field conditions. However, the use of non-destructive phenotyping entails a much more throughput evaluation without compromising selection accuracy.

### Conventional Destructive Phenotyping

**FIGURE 2:**
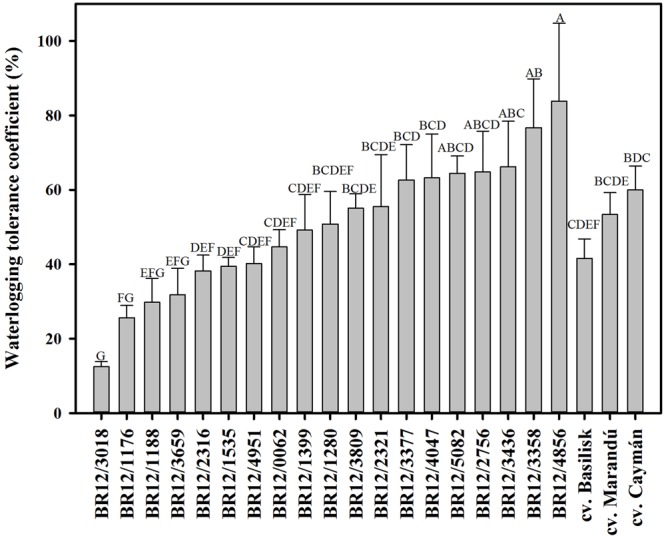
**Waterlogging tolerance coefficient (WTC) among evaluated hybrids**. Means with different letters indicate significant differences according to the Fisher-protected LSD test (*P* ≤ 0.05).

Waterlogging treatment had a negative effect on performance of all the genotypes evaluated (**Table 1**). Mean shoot biomass under waterlogged conditions decreased by 51% compared with drained conditions. The performance of hybrids under waterlogged conditions varied significantly. Two hybrids (BR12/1399; BR12/4951) showed high shoot biomass production under both waterlogged and control conditions (**Table 1**). Under waterlogged conditions, hybrids BR12/2316, BR12/1176, and BR12/3018 had significantly lower shoot biomass production in comparison with the cultivar Caymán (**Table 1**). Mean plant height under waterlogged conditions decreased by 23% compared with control conditions (**Table 1**). After 2 weeks of waterlogging, a set of hybrids (BR12/3436, BR12/1399, BR12/1535, BR12/3377, BR12/4951) were significantly taller than cultivars Basilisk and Marandu (**Table 1**). Only hybrid BR12/1316 had a significantly lower VE score than the three cultivars tested (**Table 1**). Hybrid BR12/4856, had a significantly higher waterlogging tolerance coefficient (WTC) than the commercial cultivars (**Figure 2**).

**Table 1:** Least-square means of shoot biomass (SHB), plant height after treatment and visual evaluation of symptoms in 19 *Brachiaria* hybrids and three commercial cultivars grown under control and waterlogged field conditions.

Genotype	SHB (g.plant^-1^)		Plant height (cm)		Visual evaluation (1–5)
	Control	Waterlogged	Control	Waterlogged	Waterlogged
BR12/3809	867	482	40.00	30.83	3.2
BR12/1399	1079	458	48.33	39.17	3.3
BR12/3358	526	444	36.67	30.00	3.2
BR12/1280	800	437	43.33	29.17	3.3
BR12/2321	702	426	31.67	25.00	2.8
BR12/4951	1031	416	38.33	35.00	3.2
BR12/3436	724	398	46.67	43.33	3.3
BR12/2756	561	382	40.83	33.33	3.3
BR12/3377	513	350	34.17	35.83	3.3
BR12/4856	526	346	30.35	31.67	2.8
BR12/4047	501	346	43.33	32.50	2.7
BR12/1535	790	316	41.67	35.83	3.3
BR12/0062	717	290	46.67	33.33	2.7
BR12/5082	455	290	36.67	27.50	3.2
BR12/1188	1011	221	42.50	16.67	1.8
BR12/3659	650	220	41.67	25.00	2.0
BR12/2316	384	183	24.25	15.00	1.5
BR12/1176	648	171	30.00	19.17	2.0
BR12/3018	962	125	32.50	18.33	1.8
cv. Caymán	1221	391	63.33	59.17	4.0
cv. Basilisk	774	283	32.50	19.17	2.7
cv. Marandu	586	327	25.00	20.83	3.3
Mean	728	348	38.66	29.81	2.9
CV	58	66	21.46	28.69	34.81
*R*^2^	0.27	0.35	0.59	0.65	0.35
LSD (0.05)	491	184	9.6	9.8	1.1

Harvested shoot biomass under control conditions was significantly correlated with IV (*r* = 0.58, *P* < 0.01), plant height before treatment (*r* = 0.61, *P* < 0.01), and plant height after treatment (*r* = 0.56, *P* < 0.01) (**Table 2**). Under waterlogged conditions, harvested shoot biomass was positively and significantly associated with IV (*r* = 0.58, *P* < 0.01), plant height before treatment (*r* = 0.57, *P* < 0.01), plant height after treatment (*r* = 0.80, *P* < 0.001) and visual evaluation of symptoms (*r* = 0.69, *P* < 0.01) (**Table 3**).

**Table 2:** Correlation (*r*) of least-square means of conventional destructive phenotyping traits measured under control conditions (*n* = 22).

Plant trait	Initial vigor	Plant height before treatment (cm)	Plant height after treatment (cm)	Shoot biomass (g.plant^-1^)
Initial vigor by visual scoring (1–5)	-	0.61^∗∗^	0.57^∗∗^	0.58^∗∗^
Plant height before treatment (cm)	0.61^∗∗^	-	0.94^∗∗∗^	0.61^∗∗^
Plant height after treatment (cm)	0.57^∗∗^	0.94^∗∗∗^	-	0.56^∗∗^
Shoot biomass (g.plant^-1^)	0.58^∗∗^	0.61^∗∗^	0.56^∗∗^	-

*Pearson’s correlation coefficients are indicated with their statistical significance as follows: ^∗∗^*P* ≤ 0.01; ^∗∗∗^P ≤ 0.001.*

**Table 3:** Correlation (*r*) of least-square means of conventional destructive phenotyping traits measured under waterlogged conditions (*n* = 22).

Plant trait	Plant vigor	Plant height before treatment	Plant height after treatment	Shoot biomass	Visual evaluation of symptoms	Green leaf biomass	Dead leaf biomass	WTC
Plant vigor by visual scoring (1–5)	-	0.38 ns	0.41^∗^	0.58^∗∗^	0.63^∗∗^	0.60^∗∗^	0.42^∗^	0.38 ns
Plant height before treatment (cm)	0.38 ns	-	0.84^∗∗∗^	0.57^∗∗^	0.18 ns	0.50^∗^	0.52^∗∗^	0.19 ns
Plant height after treatment (cm)	0.41^∗^	0.84^∗∗∗^	-	0.80^∗∗∗^	0.50^∗^	0.78^∗∗^	0.63^∗∗^	0.50^∗^
Shoot biomass (g.plant^-1^)	0.58^∗∗^	0.57^∗∗^	0.80^∗∗∗^	-	0.69^∗∗∗^	0.92^∗∗∗^	0.86^∗∗∗^	0.58^∗∗^
Visual evaluation of symptoms (1–5)	0.63^∗∗^	0.18 ns	0.50^∗^	0.69^∗∗∗^	-	0.75^∗∗∗^	0.43^∗^	0.64^∗∗^
Green leaf biomass (g.plant^-1^)	0.60^∗∗^	0.50^∗^	0.78^∗∗^	0.92^∗∗∗^	0.75^∗∗∗^	-	0.59^∗∗^	0.66^∗^
Dead leaf biomass (g.plant^-1^)	0.42	0.52^∗∗^	0.63^∗∗^	0.86^∗∗∗^	0.43^∗^	0.59^∗∗^	-	0.33 ns
Waterlogging tolerance coefficient (%)	0.38 ns	0.19 ns	0.50^∗^	0.58^∗∗^	0.64^∗∗^	0.66^∗^	0.33 ns	-

*Pearson’s correlation coefficients are indicated with their statistical significance as follows: ns, non-significant (*P* > 0.05); ^∗^*P* ≤ 0.05; ^∗∗^*P* ≤ 0.01; ^∗∗∗^*P* ≤ 0.001.*

### NDVI and Digital Image Processing for Non-destructive Phenotyping

We evaluated the feasibility of simple and non-destructive phenotyping by comparing it with conventional, more laborious and expensive phenotyping methods. Non-destructive image analysis of projected green area before starting treatment (PGA1) and projected green area after treatment (PGA2) were positively and linearly correlated with harvested shoot biomass among genotypes evaluated under control conditions (*r* = 0.94, *P* < 0.001 and *r* = 0.84, *P* < 0.001, respectively) (**Table 4**). Likewise, no strong correlation was found neither between PGA2 and IV (*r* = 0.52, *P* < 0.01) nor between PGA2 and both values of plant height before treatment (*r* = 0.51, *P* < 0.05) and plant height after treatment (*r* = 0.51, *P* < 0.05) (**Table 4**). NDVI values were not significantly correlated with either PGA1 (*r* = 0.40, *P* > 0.05) or with IV (*r* = 0.34, *P* > 0.05) under control conditions (**Table 4**). Plant height before treatment and plant height after treatment under control conditions had highly significant correlations with NDVI values (*r* = 0.70, *P* < 0.001 and *r* = 0.76, *P* < 0.001, respectively) (**Table 4**).

**Table 4:** Correlation (*r*) of least-square means of non-destructive phenotyping traits measured under control conditions (*n* = 22).

Plant traits	Projected green area before treatment (cm^2^.plant^-1^)	Projected green area after treatment (cm^2^.plant^-1^)	Normalized difference vegetation index
Initial vigor (1–5)	0.59^∗∗^	0.52^∗∗^	0.34 ns
Plant height before treatment (cm)	0.55^∗∗^	0.51^∗^	0.70^∗∗∗^
Plant height after treatment (cm)	0.50^∗∗^	0.51^∗^	0.76^∗∗∗^
Shoot biomass (g.plant^-1^)	0.94^∗∗∗^	0.84^∗∗∗^	0.44^∗^
Projected green area before treatment (cm^2^.plant^-1^)	-	0.90^∗∗∗^	0.40 ns
Projected green area after treatment (cm^2^.plant^-1^)	0.90^∗∗^	-	0.41^∗^
Normalized difference vegetation index	0.40 ns	0.41^∗^	-

*Pearson’s correlation coefficients are indicated with their statistical significance as follows: ns, non-significant (*P* > 0.05); ^∗^*P* ≤ 0.05; ^∗∗^*P* ≤ 0.01; ^∗∗∗^*P* ≤ 0.001.*

**FIGURE 3:**
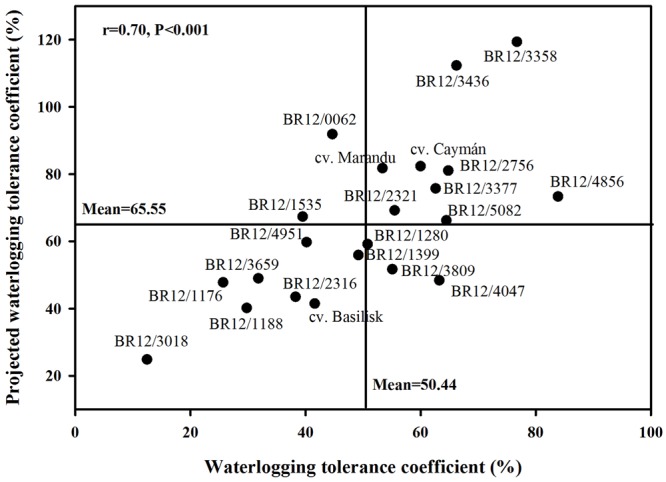
**Relationship between waterlogging tolerance coefficient and projected waterlogging tolerance coefficient**.

Under waterlogging conditions, a significant positive correlation was found between the dead and green leaf area predicted through image analysis and destructively measured dead or green leaf biomass (*r* = 0.92, *P* < 0.001 and *r* = 0.88, *P* < 0.001, respectively) (**Table 5**). Normalized difference vegetation index values were also significantly associated with total shoot biomass (*r* = 0.84, *P* < 0.001), green leaf biomass (*r* = 0.85, *P* < 0.001), plant height after treatment (*r* = 0.75, *P* < 0.001), visual evaluation of symptoms (*r* = 0.85, *P* < 0.001) and projected green area after waterlogging using digital photography (*r* = 0.85, *P* < 0.001) (**Table 5**). There was no correlation between plant vigor before treatment and projected dead area (*r* = 0.38) (**Table 5**). Plant height before starting waterlogging treatment was significantly positively related to projected green area before waterlogging treatment (*r* = 0.62, *P* < 0.01). Similarly, plant height after 2 weeks of waterlogging was significantly correlated with projected green area after waterlogging treatment (*r* = 0.73, *P* < 0.001) (**Table 5**). Visual evaluation of symptoms after waterlogging treatment were associated with projected green area after treatment (*r* = 0.71, *P* < 0.001) (**Table 5**). A significant correlation was found (*r* = 0.7, *P* < 0.001) between destructively measured WTC and projected WTC calculated from digital images (**Figure 3**).

**Table 5:** Correlation (*r*) of least-square means of non-destructive phenotyping traits measured under waterlogged conditions (*n* = 22).

Plant traits	Projected green area before treatment (cm^2^.plant^-1^)	Projected green area after treatment (cm^2^.plant^-1^)	Projected dead area (cm^2^.plant^-1^)	Normalized difference vegetation index
Initial vigor (1–5)	0.72^∗∗∗^	0.56^∗∗^	0.38 ns	0.56^∗∗^
Plant height before treatment (cm)	0.62^∗∗^	0.50^∗^	0.50^∗^	0.47^∗^
Plant height after treatment (cm)	0.59^∗∗^	0.73^∗∗∗^	0.55^∗∗^	0.75^∗∗∗^
Shoot biomass (g.plant^-1^)	0.78^∗∗^	0.87^∗∗∗^	0.74^∗∗∗^	0.84^∗∗∗^
Green leaf biomass (g.plant^-1^)	0.67^∗∗∗^	0.88^∗∗∗^	0.46^∗^	0.85^∗∗∗^
Dead leaf biomass (g.plant^-1^)	0.73^∗∗∗^	0.64^∗∗∗^	0.92^∗∗∗^	0.62^∗∗^
Visual evaluation of symptoms (1–5)	0.35 ns	0.71^∗∗∗^	0.38 ns	0.85^∗∗∗^
Projected green area before treatment (cm^2^.plant^-1^)	-	0.64^∗∗∗^	0.63^∗∗∗^	0.52^∗∗^
Projected green area after treatment (cm^2^.plant^-1^)	0.64^∗∗∗^	-	0.68^∗∗∗^	0.85^∗∗∗^
Projected dead area (cm^2^.plant^-1^)	0.63^∗∗∗^	0.68^∗∗∗^	-	0.57^∗∗^
Normalized difference vegetation index	0.52^∗∗^	0.85^∗∗∗^	0.57^∗∗^	-

*Pearson’s correlation coefficients are indicated with their statistical significance as follows: ns, non-significant (*P* > 0.05); ^∗^*P* ≤ 0.05; ^∗∗^*P* ≤ 0.01; ^∗∗∗^*P* ≤ 0.001.*

## Discussion

The results of this study demonstrate that NDVI and image-based phenotyping for non-destructive estimation of plant performance was useful to identify differences in shoot biomass production and waterlogging tolerance among *Brachiaria* genotypes under field conditions. This phenotyping methodology just requires the use of a regular Red Green Blue (RGB) camera, a static frame and the free ImageJ software, making implementation easily achievable and user friendly. Fifty plants were photographed per hour and images were analyzed in less than 10 min. Although waterlogged plots made photograph collection difficult, this analysis was done more efficiently compared with conventional phenotyping. With only few modifications in the code, our processing program could also be used to phenotype different plants under different field and stress conditions. The main constraint of imaging in the field is the ever changing intensity and spectral composition of solar irradiance, making the pixel analysis comparison inconsistent between different pictures (Joalland et al., 2016). However, by selecting an adequate color threshold of different regions of interest we were able to address this problem.

### Conventional Destructive Phenotyping

Waterlogged soil has a deleterious effect in *Brachiaria* growth. Major effects of waterlogging in *Brachiaria* includes decrease in water and nutrient uptake, stomatal closure, reduction of photosynthetic activity, stunted growth and senescence, often leading to plant death (Dias-Filho, 2006). In the present study, significant differences in harvested shoot biomass after waterlogging treatment were found among the genotypes evaluated (**Table 1**). Differences in biomass production under waterlogging conditions have previously been reported in *Brachiaria* genotypes (Dias-Filho, 2002). The advanced hybrid selections evaluated in this study showed comparable or even higher shoot biomass production under waterlogging conditions than the commercial cultivars (**Table 1**). These outstanding hybrids should be an interesting source of alleles to further exploit through breeding.

Phenotypic differences in plant height after waterlogging treatment were found among the evaluated plants (**Table 1**). Taller plants have an advantage over short plants under waterlogging conditions, as more water is required to fully cover the plants. It is widely known that plants respond to flooding or submergence conditions by growing hyponastically or changing growth habit and leaf angle (reviewed by Polko et al., 2011) as mechanisms to escape the anoxic environment. Therefore, plants with an erect growth habit might be more resistant to waterlogging conditions. Despite the mean plant height decreased under waterlogging conditions, we found a significant and strong correlation between plant height and shoot biomass under waterlogging conditions (*r* = 0.80, *P* < 0.001) (**Table 3**). This suggested that plants that were able to grow hyponastically tended to produce more biomass under waterlogging conditions. This finding suggests that plant height could be used as a simple trait in screening schemes aimed at selecting waterlogging tolerant grasses.

The WTC represents a calculation of the biomass produced under waterlogging against that produced under control conditions, and has been employed as a practical screening method to test waterlogging tolerance in maize (Liu et al., 2010). One hybrid (BR12/4856) had significantly superior WTC compared to the three commercial cultivars (**Figure 2**). However, this hybrid was not among the best performers in terms of overall shoot biomass produced under waterlogging conditions (**Table 1**), suggesting that its high WTC was influenced by its reduced growth under control conditions. Small plants require less metabolic demands for maintaining growth in comparison with bigger plants, thus it would represent a competitive advantage under waterlogging conditions. Similarly, a significant and strong correlation between shoot biomass and dead leaf biomass (*r* = 0.86, *P* > 0.0001) under waterlogging condition was found (**Table 3**), indicating that plants showing high biomass also had more proportion of dead leaves. Genotypes showing high WTC also had high projected WTC (**Figure 3**), indicating the feasibility of image analysis as a phenotyping tool. Nonetheless, no substantial differences between evaluated hybrids were found suggesting that calculation of WTC is not efficient for screening purposes in *Brachiaria*.

### NDVI and Digital Image Processing for Non-destructive Phenotyping

In the present field study, a strong correlation between analysis of regular RGB images and harvested aboveground biomass was found (**Tables 4** and **5**). The correlations between projected green and dead shoot area obtained from digital photographs of plants subjected to 2 weeks of waterlogging treatment and destructively harvested green and dead leaf biomass were highly positive and significant (*r* = 0.88, *P* < 0.0001 and *r* = 0.92, *P* < 0.0001, respectively) (**Table 5**), indicating the potential use of this approach as non-destructive phenotyping tool. This approach could also be used to determine the negative effect of waterlogging and could provide an estimate of acclimation ability -or lack of it-, as the stress continues. In a field experiment, Lootens et al. (2016) found a strong correlation (*r* = 0.82) between leaf growth predicted by digital photography and its respective destructive measurement in a *Lolium perenne* germplasm panel. Other studies in controlled greenhouse conditions have found similar correlations between areas estimated by image analysis and harvested biomass (Tackenberg, 2007; Rajendran et al., 2009; Fanourakis et al., 2014; Hairmansis et al., 2014; Joalland et al., 2016).

Although plants under control conditions were vigorous with a considerable amount of overlapping leaves (**Figure 1**), the correlation between projected green area and harvested shoot biomass was also highly positive and significant (*r* = 0.84, *P* < 0.001) (**Table 4**). This indicates that pictures collected with a regular 13 megapixels’ camera located 1.5 m above the plants give adequate resolution for phenotyping purposes. Image analysis of vigorous plants with overlapping leaves can lead to underestimations of projected areas (Fanourakis et al., 2014; Joalland et al., 2016). To address this problem, Tessmer et al. (2013) have proposed a functional analysis involving plant area estimation, growth modeling and analysis which takes overlapping leaves into account in small rosette plants. In the same way, image analysis accuracy should be improved by taking into account plant area and plant age (Golzarian et al., 2011).

Under waterlogged conditions we found significant correlations among projected green area, shoot biomass and green leaf area. NDVI values were also well-correlated with shoot biomass and green leaf biomass. Thus non-destructive phenotyping through analysis of digital photographs is a more accurate method to estimate total shoot biomass and green leaf biomass than visual estimation. As a consequence of waterlogging, all of the evaluated plants showed green and dead leaves, hindering the conventional harvest and visual scoring method. In this sense, automation of phenotyping by using complementary tools such as NDVI and image analysis offers potential benefits in labor savings and accuracy because they are not affected by human fatigue or bias. Non-destructive phenotyping through image analysis could also be used to quantify the proportion of aboveground biomass affected by waterlogging in early stages. This in turn could give information about which species can be targeted to be planted in zones with different periods of waterlogging. In the same way, analysis of growth rate under waterlogging conditions could also be performed using the proposed phenotyping technique. The advantages of image analysis are that the measurements obtained are quantitative, non-destructive, rapid, and can be used as a proxy for forage yield (Hairmansis et al., 2014). Digital photography could be used as a powerful tool for increasing selection accuracy by increasing the number of replicates evaluated and could also be applied to large mapping populations to identify genes underlying variation in senescence under stress conditions (Furbank and Tester, 2011). Thus the use of non-destructive phenotyping approaches should facilitate rapid and reliable field evaluation of a greater number of genotypes without compromising selection accuracy of a traditional visual evaluation and destructive type of analysis. This in turn will support the on-going *Brachiaria* breeding efforts and accelerate genetic gain in improving forage yield under waterlogging and also other abiotic stress factors.

## Conclusion

The high correlation observed in the present study between harvested shoot biomass and projected shoot area confirm the reliability of the non-destructive approach as a powerful tool of phenotyping to identify stress tolerant hybrids. The image processing method proposed in this study discriminated between green leaves, dead leaves and soil and was found to correlate with plant performance under waterlogged conditions. NDVI was successfully used as a complimentary measurement well-correlated to plant height. Both approaches could be applied to monitor changes in plant growth and senescence under waterlogged conditions over time. The application of this methodology could serve as a screening method specially in remote areas without access to appropriate facilities to perform conventional screening. Even though our methodology and code for image analysis was sufficiently robust to identify phenotypes under field conditions, further characteristics could be added. A side-view camera attached to the frame could improve biomass prediction and quantify plant height. Furthermore, statistical analysis could be incorporated to the code aimed to facilitate phenotyping analysis and plant identification. Further work is needed to test the suitability of this method in a set of tolerant plants in order to assess its aptitude to detect small differences between waterlogged plants as well as differences under other stresses.

## Author Contributions

JJ, JC, MW, JM, and IR designed the experiment and contributed to data interpretation. JJ and JC collected field data. JJ, JC, LL, JG, and MF analyzed images. JJ, JC, MW, and IR wrote the paper. All authors read and approved the final manuscript.

## Conflict of Interest Statement

The authors declare that the research was conducted in the absence of any commercial or financial relationships that could be construed as a potential conflict of interest.
